# DnmA and FisA Mediate Mitochondria and Peroxisome Fission, and Regulate Mitochondrial Function, ROS Production and Development in *Aspergillus nidulans*

**DOI:** 10.3389/fmicb.2020.00837

**Published:** 2020-05-04

**Authors:** Verónica Garrido-Bazán, Juan Pablo Pardo, Jesús Aguirre

**Affiliations:** ^1^Departamento de Biología Celular y del Desarrollo, Instituto de Fisiología Celular, Universidad Nacional Autónoma de México, Mexico City, Mexico; ^2^Posgrado en Ciencias Biológicas, Unidad de Posgrado, Universidad Nacional Autónoma de México (UNAM), Mexico City, Mexico; ^3^Departamento de Bioquímica, Facultad de Medicina, Universidad Nacional Autónoma de México, Mexico City, Mexico

**Keywords:** mitochondrial dynamics, mitochondrial inheritance, mitoflash, cell differentiation, mitochondrial fission

## Abstract

The dynamin-like protein Drp1 and its receptor Fis-1 are required for mitochondria and peroxisome fission in animal and yeast cells. Here, we show that in the fungus *Aspergillus nidulans* the lack of Drp1 and Fis-1 homologs DnmA and FisA has strong developmental defects, leading to a notable decrease in hyphal growth and asexual and sexual sporulation, with some of these defects being aggravated or partially remediated by different carbon sources. Although both DnmA and FisA, are essential for mitochondrial fission, participate in peroxisomal division and are fully required for H_2_O_2_-induced mitochondrial division, they also appear to play differential functions. Despite their lack of mitochondrial division, *ΔdnmA* and *ΔfisA* mutants segregate mitochondria to conidiogenic cells and produce viable conidia that inherit a single mitochondrion. During sexual differentiation, *ΔdnmA* and *ΔfisA* mutants develop fruiting bodies (cleistothecia) that differentiate excessive ascogenous tissue and a reduced number of viable ascospores. *ΔdnmA* and *ΔfisA* mutants show decreased respiration and notably high levels of mitochondrial reactive oxygen species (ROS), which likely correspond to superoxide. Regardless of this, *ΔdnmA* mutants can respond to an external H_2_O_2_ challenge by re-localizing the MAP kinase-activated protein kinase (MAPKAP) SrkA from the cytoplasm to the nuclei. Our results show that ROS levels regulate mitochondrial dynamics while a lack of mitochondrial fission results in lower respiration, increased mitochondrial ROS and developmental defects, indicating that ROS, mitochondrial division and development are critically interrelated processes.

## Introduction

Our work has been oriented to demonstrate that ROS play critical signaling roles in cell differentiation (Hansberg and Aguirre, 1990; Aguirre et al., 2005; Mendoza-Martinez et al., 2017; Mendoza-Martínez et al., 2019). In one approach, we have used the filamentous fungi *Aspergillus nidulans* to study the mechanisms by which cells perceive and respond to external ROS. In this and other fungi, the SakA-MpkC stress MAPK pathway plays crucial roles in responding to multiple types of stress, including oxidative stress (Kawasaki et al., 2002; Lara-Rojas et al., 2011; Garrido-Bazan et al., 2018). During the course of that work, we found that the MAPKAP-kinase SrkA is part of this pathway and that in response to external H_2_O_2_ it translocates from the cytoplasm to either the nucleus or the mitochondria, depending on the presence of the upstream MAPK SakA. Notably, under these conditions, mitochondria underwent extensive fragmentation, consistent with the induction of mitochondrial division by H_2_O_2_ (Jaimes-Arroyo et al., 2015).

Because mitochondrial replication depends on pre-existing organelles, mitochondrial division is a highly regulated process, key to many cellular activities such as cell division, autophagy and mitophagy (Horbay and Bilyy, 2016; Burman et al., 2017). Mitophagy is critical to maintain mitochondrial quality by disposing damaged mitochondrial components, including mDNA. Indeed, the lack of mitochondrial fission results in mice embryonic lethality (Wakabayashi et al., 2009) and several human pathologies are related to defects in mitochondrial dynamics (Poole et al., 2008; Trevisan et al., 2018).

The dynamin-related protein Dnm1, known as Drp1 in animal cells, is a GTPase that assembles on the mitochondrial surface and is essential for mitochondrial division (Bleazard et al., 1999). In yeast and other fungi, Dnm1 is recruited to mitochondria by the adaptor protein Fis1 along with adaptors Mdv1 and Caf4 (Tieu and Nunnari, 2000; Tieu et al., 2002; Griffin et al., 2005) and the same proteins, including Dnm1, are also required for peroxisome fission (Motley et al., 2008). In addition, mitochondria and peroxisomes share functions in the beta-oxidation of fatty-acids and both organelles are a source of ROS (Wanders et al., 2016).

The fungus *A. nidulans* constitutes an excellent model system to study cell biology processes during growth and cell differentiation, as it can undergo both asexual and sexual development. Asexual development (conidiation) is better understood and it involves the formation of complex conidiophore structures, which after growing a fixed length toward the air, develop a multinucleated vesicle, from which uninucleated cells called metulae emerge, and these in turn differentiate the conidiogenic cells called phialides. Phialides undergo a series of mitotic divisions to generate long chains of uninucleated conidia, which represent the most important dispersal strategy for this fungus (Timberlake and Clutterbuck, 1994). Sexual development involves the differentiation of an ascogenous tissue that gives rise to asci and ascospores, which is surrounded by a network of sterile hyphae that develops into the melanized cleistothecial wall or peridium. In turn, cleistothecia are surrounded by globose cells called Hülle cells, often considered as nurse cells (Champe et al., 1994; Shon and Yoon, 2002).

Here we decided to study the roles that Drp1 and Fis1 homologs play in *A. nidulans* as an approach to understand the induction of mitochondrial division by H_2_O_2_ and the roles of mitochondrial dynamics in fungal stress responses and development.

## Materials and Methods

### Strains, Media, and Growth Conditions

*Aspergillus nidulans* strains used in this work are listed in Supplementary Table S1. All strains were grown at 37°C in glucose minimal nitrate medium (MM) (Hill and Käfer, 2001), plus supplements.

The different gene-deletion constructs were produced by double-joint PCR (Yu et al., 2004) using genomic DNA as template and different primer combinations. To delete the *dnmA* gene (ANID_08874), PCR fragments were generated with primers 5′ForDnm2/5′RevDnm2 and 3′ForDnm2/3′RevDnm2. *Aspergillus fumigatus pyrG* (*AfpyrG*) marker was amplified with primers pyrGFor and pyrGRev, using plasmid PFNO3 as template (Nayak et al., 2006). These three fragments were purified, mixed and used in a fusion PCR with primers 5′NestDnm2 and 3′NestDnm2. The final 3609 bp dnmA–AfpyrG–dnmA cassette was purified and used to transform *A. nidulans* strain A1155 by electroporation (Sanchez and Aguirre, 1996; Sanchez et al., 1998). Eight PyrG^+^ transformants were obtained, analyzed by PCR to confirm *dnmA* elimination and transformant TVG1 was chosen for additional experiments (Supplementary Figure S1). Strain TVG1 was crossed to strain TRV1 to label mitochondria and eliminate the *kuA* deletion. From this cross, strain CVG1 was selected for further experiments.

A similar strategy was used to delete the *fisA* gene (ANID_06225), using primers 5′ForFis2/5′RevFis2 to produce *fisA* 5′ region and 3′ForFis2/3′RevFis2 for *fisA* 3′ region. *A. fumigatus pyrG* marker was amplified, as above. These 3 fragments were purified and mixed with primers 5′NestFis2 and 3′NestFis2 to produce a final 2478 bp fisA–AfpyrG–fisA product, which was used to transform *A. nidulans* strain A1155 by electroporation. Four PyrG^+^ transformants were obtained and analyzed by PCR to confirm *fisA* elimination (Supplementary Figure S2). Strain TVG2 was chosen and crossed to strain TRV1 to mark mitochondria and get rid of the *kuA* deletion. From this cross, strain CVG2 was selected for further experiments.

To label mitochondria, we transformed wild-type strain CLK43 with plasmid pPABLE, which confers resistance to phleomycin. pPABLE contains the DNA sequence encoding the 79 aa pre-mitochondrial sequence of *Podospora anserina* ATP7-9 fused to mCherry and expressed from *P. anserina gpdA* promotor (Navarro-Espíndola et al., 2020). The presence of labeled mitochondria was confirmed by epifluorescence microscopy, and strain TRV1 was chosen for additional experiments.

### Oxygen Consumption

Oxygen consumption was determined by high-resolution respirometry, using the Oroboros oxygraphy-2k (Oroboros Instruments, Innsbruck, Austria) calibrated at 30°C. Spores (1X10^8^) from strains TRV1 (WT), CVG1 (*ΔdnmA*), CVG2 (*ΔfisA*), and CVG3 (*ΔdnmA ΔfisA*) were inoculated in 200 ml of liquid minimal medium and incubated at 37 °C with shaking for 4 h. After this, spores were collected, washed three times with ice-cold MM without glucose, collected again by centrifugation and weighted. Spores were finally resuspended in MM without glucose at 0.1 mg/μl and maintained on ice until used. 2.5 mg of spores were used for every determination and added to the respirometry chamber containing 2 ml of MM without glucose. Routine respiration was measured by adding glucose (1%), and CCCP (10 μM) was used to determine maximum respiration. Three independent determinations were made. Data were analyzed using DatLab6 software (Oroboros Instruments).

### Microscopy

Fluorescence microscopy images were captured *in vivo*. To study mitochondria morphology in growing hyphae, 14 h grown mycelia was treated with or without 5 mM H_2_O_2_ for 20 min and then observed using a Zeiss LSM800 inverted laser scanning confocal microscope using a Plan Apochromat 63_/1.4 oil immersion objective and the 561 nm laser line. Images were processed using software ZEN 2012 (Carl Zeiss, Jena, Germany).

### Mitochondrial ROS Detection

MitoSOX Red (Invitrogen Waltham, MA, United States) was used to measure mitochondrial ROS. A 5 mM stock solution prepared in DMSO was maintained frozen and diluted with water to a final 5 μM working concentration. This solution was used to cover sections of solid medium containing growing mycelia, during 20 min at 37°C. After this, the MitoSOX Red solution was removed, the mycelia rinsed two times with water and immediately observed using confocal microscopy. For Mito TEMPO/MitoSOX treatments, sections of solid medium containing growing mycelia were covered with a 100 μM solution of mito TEMPO (Merck, KGaA, Darmstadt, Germany) during 2 h at 37°C. After this, the mito TEMPO solution was removed, mycelia washed and then covered with a 5 μM MitoSOX Red solution during 20 min at 37°C and immediately observed using confocal microscopy. Images were processed using Software ZEN 2012 (Carl Zeiss, Jena, Germany).

### Complementation of *ΔdnmA* and *ΔfisA* Mutants

For *ΔdnmA* and *ΔfisA* mutant complementation the corresponding genes were cloned in plasmid pEM-03 and used to transform the corresponding mutants. Briefly, *A. nidulans* genomic DNA was used as template to amplify by PCR *dnmA* and *fisA* DNA fragments, using primer pairs CV3-CV4 and CV5-CV6, respectively. Using In-phusion and vector primers CV1-CV2, these fragments, containing 1000 bp upstream and 500 bp downstream of each gene ORF were cloned into plasmid pEM-03 (E. Martínez and J. Aguirre, unpublished), which includes *A. nidulans argB* gene as selective marker. Resulting plasmids pVDnmA and pVFisA were used to transform strains CVG36 and CVG37 and ArgB^+^ transformants CVG38 and CVG39 were selected for additional experiments.

## Results

### The Dynamin-Like Protein DnmA and Its Putative Receptor FisA Regulate *A. nidulans* Growth and Development

To determine Drp1 and Fis-1 functions in *A. nidulans*, we first used yeast Dnm1 (Drp1) and Fis-1 (Fis1) proteins to perform a BLAST search against the *AspGD* database (Cerqueira et al., 2014). Protein AN8874 (794 amino acids) was identified as the Dnm1 homolog (56% identity) and named here as DnmA. Protein AN6225 (153 amino acids) was identified as the Fis1 homolog (40% identity) and renamed here as FisA. Then, we used double-joint PCR to generate *dnmA* and *fisA* deletion constructs, based on the *AfpyrG* gene as selective marker, which were used to transform strains A1155 or TRJ7. Transformants TVG1 (*ΔdnmA*) and TVG2 (*ΔfisA*) were confirmed by PCR (Supplementary Figures S1, S2, respectively). Strains TVG1 and CVG2 were crossed to obtain *ΔdnmA ΔfisA* double mutant CVG3, which was also confirmed by PCR (Supplementary Figure S3). In these and other sexual crosses *ΔdnmA* and *ΔfisA* phenotypes co-segregated always with the *AfpyrG* marker. In addition, we performed gene complementation experiments showing that *ΔdnmA* and *ΔfisA* mutant phenotypes were complemented by plasmids containing the respective wild type genes (Supplementary Figure S4).

**FIGURE 1:**
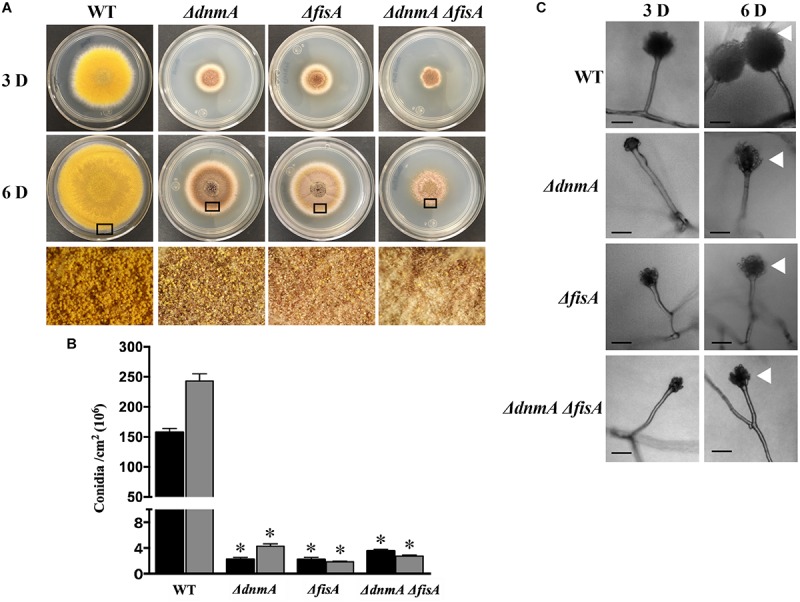
*dnmA* and *fisA* genes are required for normal growth and asexual development. **(A)** Conidia (1x10^4^) from strains TRV1 (WT), CVG1 (*ΔdnmA*), CVG2 (*ΔfisA*), and CVG3 (*ΔdnmA ΔfisA*) were inoculated on supplemented MM plates and incubated at 37°C for 3 (upper panel) or 6 (middle panel) days (D). Lower panels show enlargements from 6-day colony borders. **(B)** Total conidiospores per colony were harvested, counted and the count divided by colony area to determine number of conidia per square centimeter. Black and gray bars represent 3 D and 6 D spore numbers, respectively. Standard deviation from three independent experiments is indicated. Data analyzed by one-way ANOVA, following Tukey’s test (^∗^*p*< 0.05). Asterisks indicate significant differences with respect to the WT strain. **(C)** Conidiophores from the indicated strains were obtained from 3 and 6-day colonies and observed under the microscope. White arrowheads point to conidiophore heads. Black bars = 20 μm.

**FIGURE 2:**
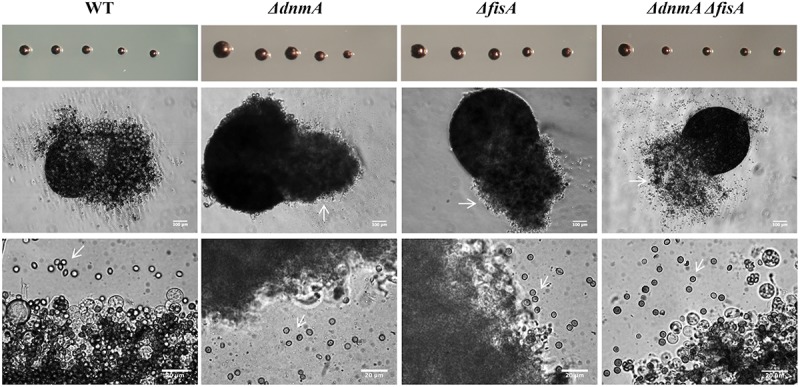
The lack of *dnmA* or *fisA* causes severe defects in sexual development. Strains TRV1 (WT), CVG1 (*ΔdnmA*), CVG2 (*ΔfisA*), and CVG3 (*ΔdnmA ΔfisA*) were induced to undergo sexual development as previously reported (Kawasaki et al., 2002). Intact (upper panel) or crushed Cleistothecia (middle and lower panels) from 8-day old cultures were isolated and observed under the microscope. White arrows point to ascogenous tissue (middle panel) and ascospores (lower panel).

As shown in Figure 1, *ΔdnmA*, *ΔfisA*, and *ΔdnmA ΔfisA* mutants produced very similar phenotypes in glucose minimal medium. They all showed a drastic reduction in both, radial growth (Figure 1A) and the production of asexual spores (Figure 1B). The decrease in conidiation was due to the production of lower number of conidiophores (Figure 1A, bottom panel) and the fact that conidiophore head size and the number of asexual spores (conidia) formed by each conidiophore were also notably reduced (Figure 1C). None of the mutants showed sensitivity to the cell-wall disturbing agent calcofluor. However, they all showed sensitivity to congo red (Supplementary Figure S5), suggesting that they all have a specific type of cell-wall defect. Notably, all three mutants developed large numbers of Hülle cells, indicating a premature initiation of sexual development (Figure 1A, lower panels and Supplementary Figure S6). On a closer inspection, it became clear that *ΔdnmA*, *ΔfisA*, and *ΔdnmA ΔfisA* mutants were not only able to develop Hülle cells but also capable to differentiate fruiting bodies or cleistothecia with normal appearance. However, when these cleistothecia were broken to release the ascospores, it became evident that *ΔdnmA* and *ΔfisA* cleistothecia produced large amounts of sterile ascogenous tissue and much lower numbers of viable ascospores, when compared to wild type cleistothecia (Figure 2 and Supplementary Figure S6).

The fact that *ΔdnmA*, *ΔfisA*, and *ΔdnmA ΔfisA* mutants show very similar phenotypes suggests that in *A. nidulans* the dynamin-like DnmA and its putative mitochondrial receptor FisA function in the same pathway and that both proteins are required for normal growth and normal asexual and sexual development.

### DnmA and FisA Are Essential for Mitochondrial Fission in Response to H_2_O_2_ and During Growth

To determine the roles of DnmA and FisA in mitochondrial fission, we first generated strain TRV1, in which mitochondria were labeled with protein mCherry. This strain was crossed to strains TVG1 (*ΔdnmA*), TVG2 (*ΔfisA*), and strain TVG1 was crossed to CVG2 to obtain *ΔdnmA*, *ΔfisA*, and *ΔdnmA ΔfisA* mutants with labeled mitochondria.

As we have previously shown that H_2_O_2_ induces mitochondrial fragmentation in *A. nidulans* (Jaimes-Arroyo et al., 2015), we wanted to test if DnmA and FisA were required for such stress response, as well as for normal mitochondrial division. For this, we first determined the lowest non-lethal concentration of H_2_O_2_ that induced mitochondrial fragmentation in a wild type strain, within 5-20 min. As shown in Supplementary Figure S7, wild type young colonies treated with 5 mM H_2_O_2_ for 5–20 min grew as well as a non-treated colony. Under these conditions, growing hyphae not treated with H_2_O_2_ display mitochondria mostly as long filaments, along with some smaller round mitochondria (Supplementary Figure S8, top panel). In sharp contrast, a 5 mM H_2_O_2_ treatment induces moderate mitochondrial fission after 5 min and extensive fission after 20 min (Supplementary Figure S8, middle and lower panels).

**FIGURE 3:**
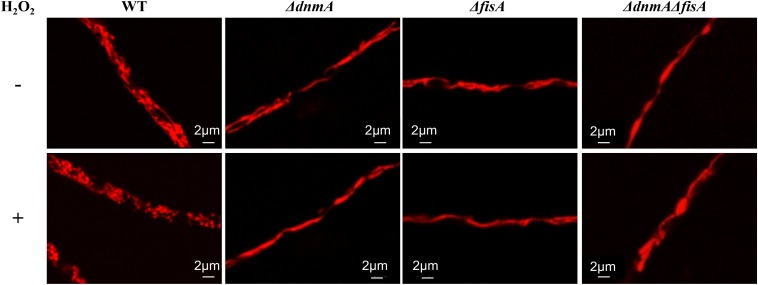
*dnmA* and *fisA* genes are essential for mitochondrial fission induced by H_2_O_2_. Mycelia from strains TRV1 (WT), CVG1 (*ΔdnmA*), CVG2 (*ΔfisA*), and CVG3 (*ΔdnmA ΔfisA*) grown for 14 h in supplemented glucose minimal medium were treated (lower panel) or not (upper panel) with 5 mM H_2_O_2_ for 20 min and then observed by using confocal microscopy.

Figure 3 shows the mitochondrial morphology of wild type, *ΔdnmA*, *ΔfisA* and *ΔdnmA ΔfisA* mutants with and without a 20 min 5 mM H_2_O_2_ treatment. As indicated, wild type mitochondria form long filaments along with some smaller mitochondria. In sharp contrast, in all three mutants mitochondria formed long and uninterrupted filaments, which were not fragmented in the presence of H_2_O_2_. These results clearly show that DnmA and FisA are essential components of the mitochondrial fission machinery in *A. nidulans* and that both mediate the mitochondrial fission induced by H_2_O_2_.

### A Lack of Mitochondrial Fission Does Not Prevent Mitochondrial Inheritance During Asexual Development

**FIGURE 4:**
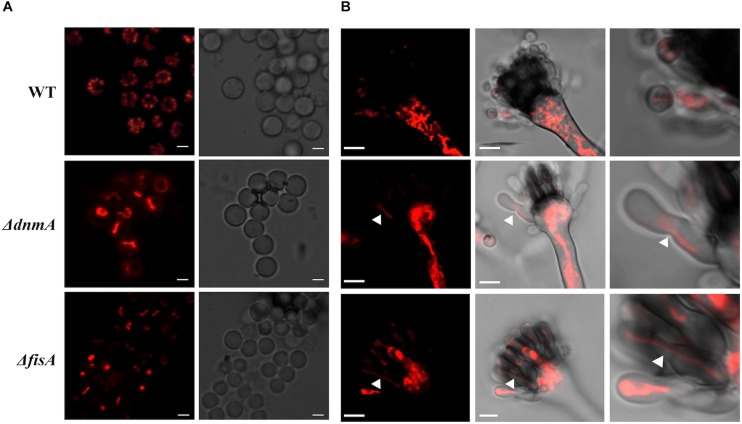
Mitochondrial inheritance during asexual development in WT, *ΔdnmA*, and *ΔfisA* strains. **(A)** Isolated conidia from strains TRV1 (WT), CVG1 (*ΔdnmA*), and CVG2 (*ΔfisA*) were observed by using confocal microscopy. **(B)** Developing conidiophores from the same strains were also observed under the same conditions. The right panels show a magnification from a section of the middle panels. White arrowheads in *ΔdnmA* and *ΔfisA* mutant panels point to mitochondrial filaments located between metulae and nascent phialide cells. White bars = 2 and 5 μm.

Mitochondrial fission is closely related to cell division, as a mechanism to secure mitochondrial distribution among dividing cells. Given the lack of mitochondrial fission observed in *ΔdnmA*, *ΔfisA* and *ΔdnmA ΔfisA* mutants, we explored mitochondrial inheritance during conidiation in mutant and wild type strains. As shown in Figure 4A, wild type conidia contain multiple individual mitochondria, while all conidia produced by *ΔdnmA* and *ΔfisA* mutants contained a larger single mitochondrion. To understand such different patterns of mitochondria inheritance, we compared mitochondrial morphology in young wild type and *ΔdnmA* and *ΔfisA* conidiophores. Wild type conidiophores displayed high levels of fissioned mitochondria at the vesicle stage, which were segregated to nascent metulae and phialides. Interestingly, the stalks and vesicles from *ΔdnmA* and *ΔfisA* conidiophores displayed a convoluted network of mitochondrial filaments, from which long mitochondrial filaments branched and moved to developing metulae and phialides, before a septum separated these cell types (Figure 4B). These results indicate that conidial development is not completed without the presence of mitochondria, and that mitochondria unable to undergo fission can still branch, move and suffer DnmA-independent fission during conidia formation, very likely by a mechanical septation-mediated process.

### DnmA and FisA Also Regulate Peroxisomal Fission

**FIGURE 5:**
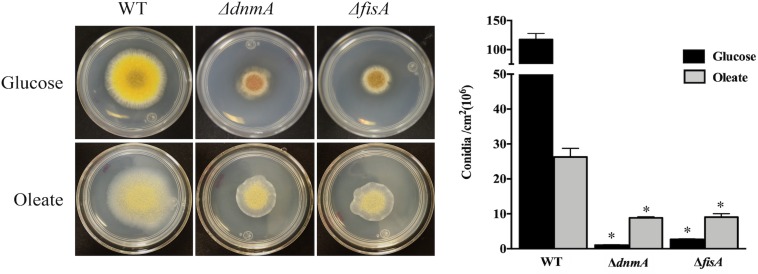
Growth and asexual sporulation of *ΔdnmA* and *ΔfisA* mutants are improved in oleate as sole carbon source. Conidia from strains RPA (WT), CVG30 (*ΔdnmA*), and CVG31 (*ΔfisA*) were inoculated on supplement MM plates containing either glucose or oleate as sole carbon source and incubated at 37°C for 3 days and photographed **(Left)** and conidia per square centimeter were determined **(Right)**. Bars indicate standard deviation from three independent experiments. Data analyzed by one-way ANOVA, following Tukey’s test (^∗^*p*< 0.05). Asterisks indicate significant differences with respect to the WT strain.

**FIGURE 6:**
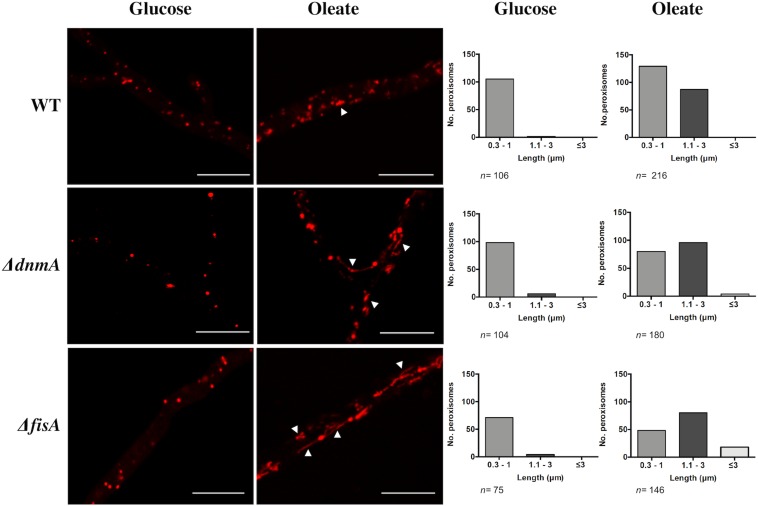
DnmA and FisA regulate peroxisomal fission. Mycelia from strains RPA (WT), CVG30 (*ΔdnmA*), and CVG31 (*ΔfisA*) grown for 48 h on supplemented MM containing either glucose or oleate as sole carbon source was observed using confocal microscope **(Left)**. Peroxisome number was determined and, according to their length, peroxisomes were classified **(Right)** in categories short (0.3–1.0 μm), medium (1.1–3.0 μm), and long (more than 3 μm). A representative experiment is shown. Arrowheads in *ΔdnmA* and *ΔfisA* mutants point to larger and filamentous peroxisomes. White bars = 10 μm.

To examine the role of DnmA and FisA in peroxisomal fission, we grew *A. nidulans* in oleate medium as sole carbon source, where peroxisome proliferation has been reported (Valenciano et al., 1998). As seen in Figure 5, both, growth and conidiation phenotypes were improved under these conditions. Indeed, in glucose *ΔdnmA* and *ΔfisA* conidiation represents about 2% of the wild type conidiation while in oleate this value increases to about 33 and 34% for *ΔdnmA* and *ΔfisA* mutants, respectively. As reported, the number and size of peroxisomes increased in oleate in the wild type strain. Although peroxisome numbers also were higher in oleate than in glucose in *ΔdnmA* and *ΔfisA* mutants, peroxisome size and shape were notably modified. Indeed, many peroxisomes displayed a filamentous shape in the *ΔdnmA* and *ΔfisA* mutants (Figure 6). However, mitochondrial morphology was not notably affected under these conditions (not shown). These results indicate that DnmA and FisA regulate peroxisomal fission and suggest that other mechanisms contribute to this process.

### Growth and Conidiation of *ΔdnmA* and *ΔfisA* Mutants Are Also Improved in Ethanol, Arabinose, and Glycerol

**FIGURE 7:**
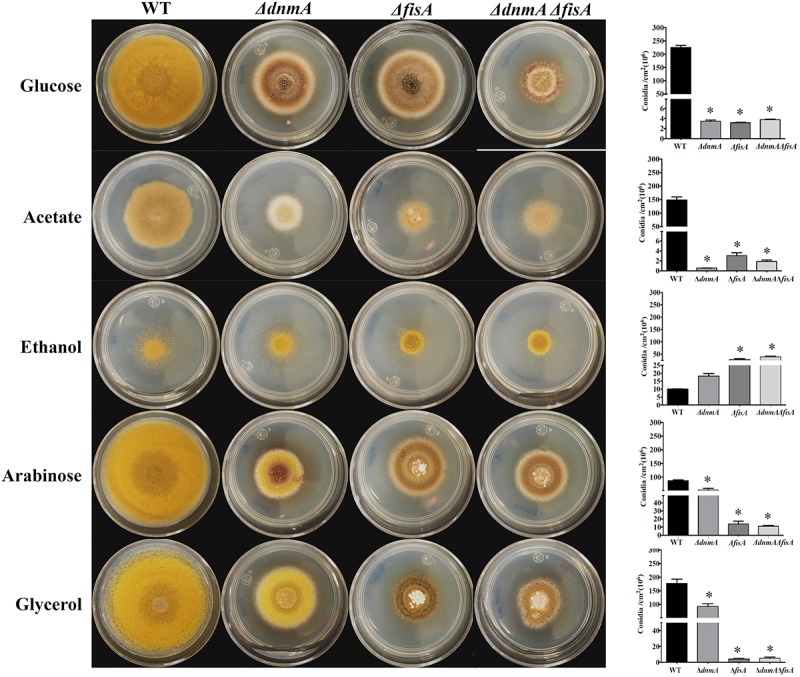
Growth and conidiation of mitochondrial fission mutants are differentially affected by different carbon sources. Conidia (1 × 10^4^) from strains TRV1 (WT), CVG1 (*ΔdnmA*), CVG2 (*ΔfisA*), and CVG3 (*ΔdnmA ΔfisA*) were inoculated on supplemented MM plates with the indicated carbon sources and incubated at 37°C for 3 days **(Left)** and conidiation was quantitated **(Right)**. Bars indicate standard deviation from three independent experiments. Data analyzed by one-way ANOVA, following Tukey’s test (^∗^*p*< 0.05). Asterisks indicate significant differences with respect to the WT strain.

After detecting that *ΔdnmA* and *ΔfisA* mutant growth and conidiation phenotypes were moderately improved in oleate as sole carbon source, we decided to test the response to other carbon sources. While phenotypes were not notably affected when mutant strains were grown on fructose or galactose (Supplementary Figure S9), clear phenotypic changes were appreciated in the other media (Figure 7). Indeed, *ΔdnmA* conidiation was reduced in acetate and increased in ethanol, arabinose, and glycerol. This effect was more prominent in glycerol, where conidiation reached about 52% of wild type conidiation. Notably, in arabinose and glycerol these changes were not observed in the *ΔfisA* mutant (Figure 7). As mitochondrial fission is not restored under these conditions (not shown), these results suggest that a lack of mitochondrial fission affects mitochondrial functions related to the utilization of different carbon sources, and that despite their common role in mitochondrial fission, DnmA, and FisA might also play different and specific functions in fungal physiology.

### The Lack of DnmA and FisA Has Effects on Respiration and Results in Increased Mitochondrial ROS Production

**FIGURE 8:**
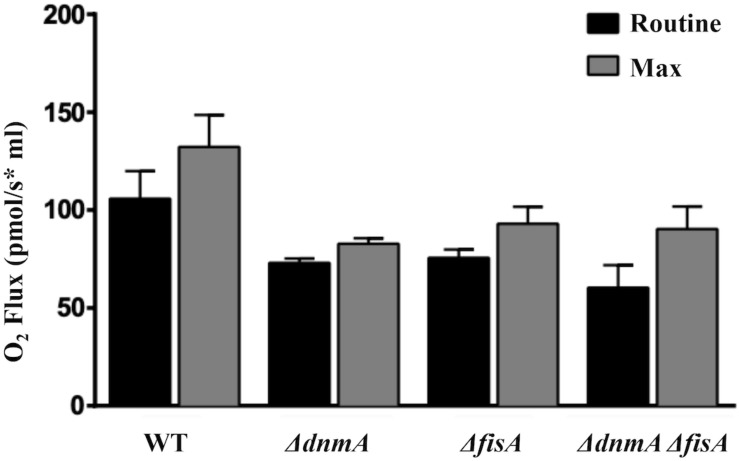
The lack of mitochondrial fission results in decreased *in vivo* respiration. Conidia from strains TRV1 (WT), CVG1 (*ΔdnmA*), CVG2 (*ΔfisA*), and CVG3 (*ΔdnmA ΔfisA*) were incubated in supplemented glucose-MM for 4 h, washed three times with ice-cold MM without glucose and maintained on ice until used for respiration measurements. Respiration was determined using an Oroboros high-resolution respirometer, as indicated in section “Materials and Methods.”

To explore if the lack of mitochondrial fission affected cell respiration and mitochondrial ROS content, we decided to compare wild type and mutant respiration *in vivo*. However, to measure respiration using mycelia is complicated by the inherent heterogeneity of hyphal age and composition (i.e., mycelial pellets, vacuolated vs. non-vacuolated regions, nuclei, and mitochondria number, etc.). Having shown that all conidia from *ΔdnmA*, *ΔfisA*, and *ΔdnmA ΔfisA* mutants inherit mitochondria in an autoregulated process, we decided to determine respiration using uninucleate conidia germinated for only 4 h. As shown in Figure 8, compared to the wild type strain *ΔdnmA*, *ΔfisA*, and *ΔdnmA ΔfisA* mutants presented about 20% reduction in routine and maximum respiration. These results indicate that the lack of mitochondrial fission has a notable but not so drastic impact on respiration.

**FIGURE 9:**
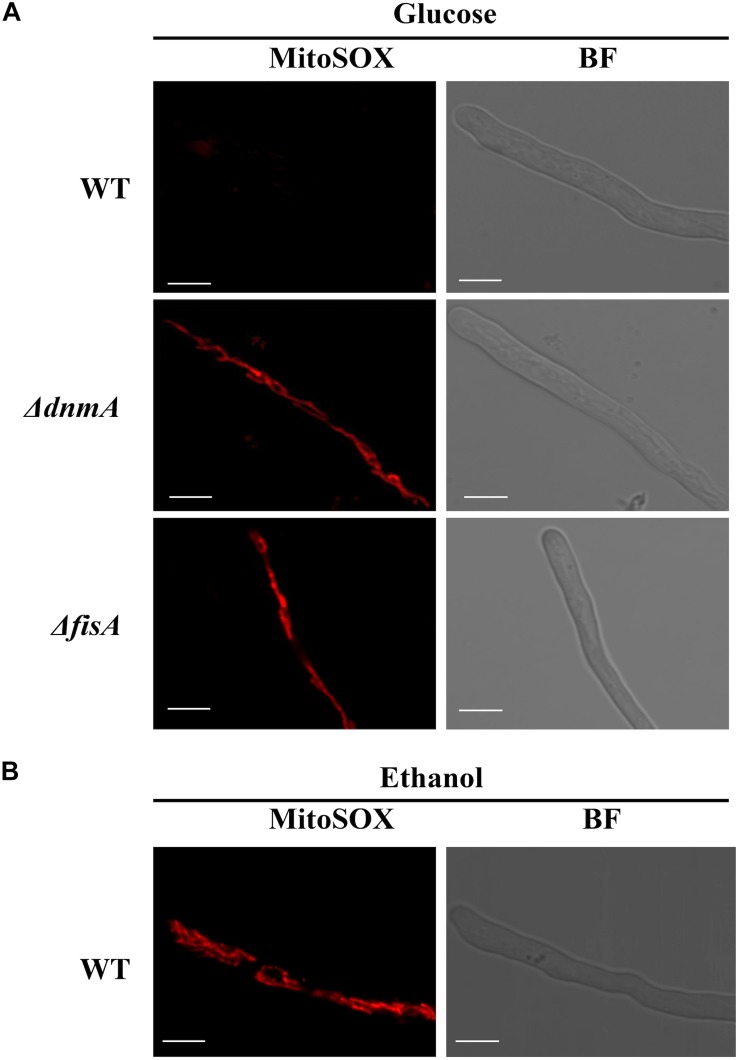
The lack of mitochondrial fission results in increased mitochondrial ROS production. **(A)** Mycelia from strains A1155 (WT), TVG1 (*ΔdnmA*), and TVG2 (*ΔfisA*) grown for 14 h in supplemented glucose minimal medium were treated with MitoSOX as indicated in Materials and Methods and then observed by using confocal microscopy. **(B)** Mycelia from strain A1155 (WT) grown for 14 h in supplemented 1% ethanol minimal medium was treated with MitoSOX as before and observed by using confocal microscopy. BF, bright field. White bars = 5 μm.

To explore the possibility that a lack of mitochondrial fission could result in increased mitochondrial ROS production, we used the compound MitoSOX as reporter of mitochondrial superoxide and other ROS (Robinson et al., 2006). As shown in Figure 9A and Supplementary Figure S4B, mitochondria from *ΔdnmA* and *ΔfisA* mutants were readily stained by MitoSOX. In sharp contrast, mitochondria from the wild type (Figure 9A) or the complemented mutant strains (Supplementary Figure S4B), were not stained under the same conditions. However, MitoSOX staining of wild type mitochondria was observed when the wild type strain was grown on ethanol as sole carbon source (Figure 9B), a condition we have shown that induces oxidative stress (Mendoza-Martinez et al., 2017).

**FIGURE 10:**
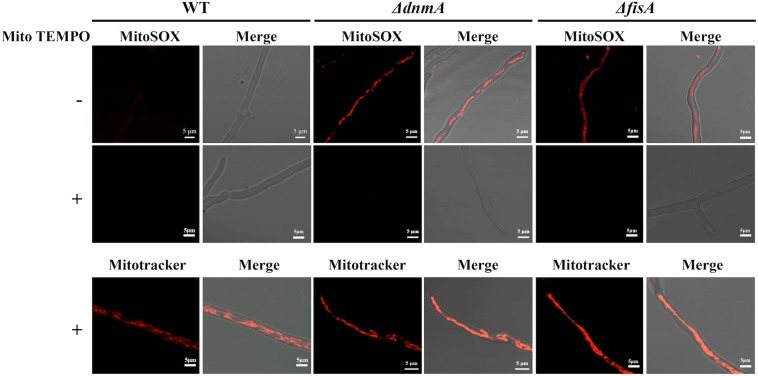
Pretreatment with Mito TEMPO prevents MitoSOX but not Mitotracker staining of *ΔdnmA* and *ΔfisA* mitochondria. Mycelia from strains 1155 (WT), TVG1 (*ΔdnmA*), and TVG2 (*ΔfisA*) grown for 14 h in supplemented glucose minimal medium were incubated with Mito TEMPO for two h at 37°C and then stained with either MitoSOX or Mitotracker, as indicated in section “Materials and Methods,” and then observed by using confocal microscopy.

Although widely used to detect superoxide in mitochondria, MitoSOX specificity has been questioned. Despite that, it is considered an ideal probe to measure mitochondrial oxidant formation (Zielonka and Kalyanaraman, 2010). To explore the nature of the mitochondrial ROS detected in our experiments, we decided to pretreat *ΔdnmA* and *ΔfisA* mutants with the mitochondrial superoxide scavenger mito TEMPO (Dikalova et al., 2010), before MitoSOX staining. As shown in Figure 10, such mito TEMPO pretreatment prevented the staining of *ΔdnmA* and *ΔfisA* mitochondria by MitoSOX but not by Mitotracker.

These results, linking the lack of mitochondrial fission to an increased accumulation of mitochondrial ROS, led us to test *ΔdnmA* and *ΔfisA* mutant sensitivity to external ROS. As shown in Supplementary Figure S10, the wild type, *ΔdnmA* and *ΔfisA* strains resulted equally sensitive to H_2_O_2_. In contrast, *ΔdnmA* and *ΔfisA* mutants were more sensitive than the wild type strain to menadione, which induces mitochondrial superoxide production. These results support a model in which the lack of mitochondrial fission results in an increased production of mitochondrial superoxide.

**FIGURE 11:**
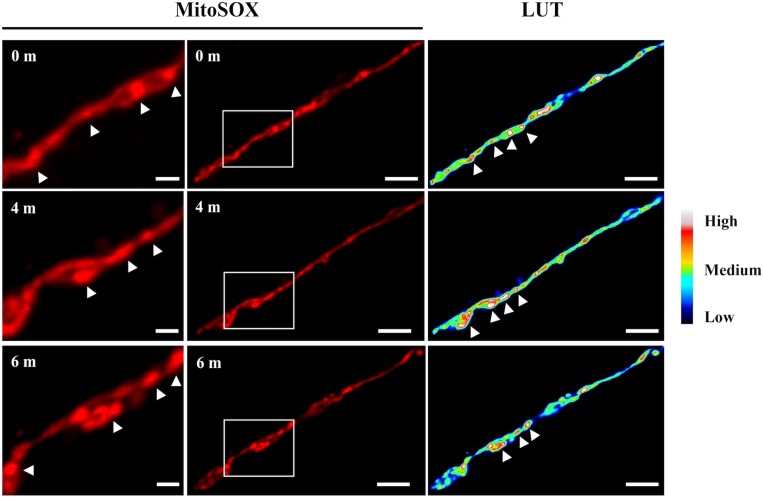
Time course observation of *ΔdnmA* mitochondria stained with MitoSOX. Mycelia from strain TVG1 (*ΔdnmA*) grown for 14 h in supplemented glucose minimal medium was stained with MitoSOX, as indicated in section “Materials and Methods,” and then illuminated at 0, 4 and 6 min and observed by using confocal microscopy (center panels). Right panels show the relative quantification of the fluorescence intensity (LUT). Left panels show a magnification of squared regions in the central panels, where higher fluorescence intensity regions are indicated by white arrowheads. White bars = 5 μm.

A time-course observation of MitoSOX staining of the *ΔdnmA* mutant shows that although entire mitochondria were stained by MitoSOX, it was possible to detect discrete and dynamic mitochondrial regions transiently showing higher fluorescence intensity (Figure 11) and the same was observed in *ΔfisA* mutants (not shown). This suggests that superoxide or other ROS are initially produced or accumulated in different mitochondrial regions and then spread to the rest of the mitochondria, in a phenomenon that is reminiscent of the mitoflashes described in animal cells (Feng et al., 2019).

### Increased Mitochondrial ROS in *ΔdnmA* Mutants Does Not Affect SrkA Cytosolic Localization

**FIGURE 12:**
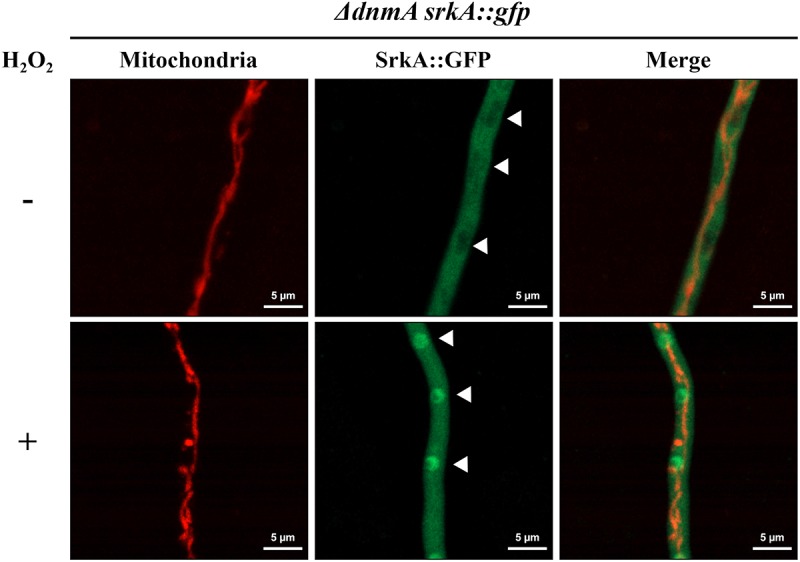
H_2_O_2_ induces SrkA nuclear localization in *ΔdnmA* mutants. Mycelia from strain CVG4 (*ΔdnmA srkA::gfp*) grown for 14 h in supplemented glucose minimal medium was treated or not with 5 mM H_2_O_2_ for 20 min and then observed by using confocal microscopy. Arrowheads point to nuclei.

Using a nuclear marker, we have previously shown that in the absence of H_2_O_2_ the MAPKAP SrkA is localized in the cytoplasm and excluded from nuclei. In contrast, H_2_O_2_ induces SrkA nuclear localization, in a process that depends on the stress MAPK SakA (Jaimes-Arroyo et al., 2015). We decided to use these changes in SrkA distribution as an assay to detect if the high ROS levels detected in mitochondria from *ΔdnmA* mutants affected cytosolic ROS levels. As shown in Figure 12, in a *ΔdnmA* mutant SrkA was distributed in the cytoplasm and excluded from discrete compartments with size and distribution consistent with nuclear structures (indicated by arrowheads), while a treatment with H_2_O_2_ induced SrkA accumulation in these structures, as it occurs in a wild type background (Jaimes-Arroyo et al., 2015). These results indicate that while non-fissioned mitochondria produce constitutive high-ROS levels, this doesn’t seem to result in constitutive high cytoplasmic ROS levels, perhaps as result of an adaptation to such chronic condition. Therefore, mitochondrial fission-defective *ΔdnmA* mutants are still able to respond to high external H_2_O_2_ levels, which might explain their lack of sensitivity to H_2_O_2_.

## Discussion

### Mitochondrial Fission Is Connected to Development and Induced by H_2_O_2_

Our results show that *A. nidulans* growing hyphal tips contain mitochondria that are mostly organized as filamentous networks. In contrast, during conidiophore development mitochondria suffer extensive division at the vesicle stage and individual conidia inherit multiple small mitochondria. We have shown that proteins DnmA and FisA are essential for mitochondrial division, partially required for peroxisome division and that mutants lacking these proteins show severe defects in growth and in asexual and sexual development, consistent with the growth and reduced conidiation defects reported before in *A. nidulans* (Leiter et al., 2016) and *A. fumigatus ΔdnmA* mutants (Neubauer et al., 2015). Despite their drastic reduction in conidiation, all *ΔdnmA* conidia inherit a single mitochondrion, indicating that mitochondria can undergo DnmA-independent fission by a mechanical septation-mediated process, which might occur not only during conidiation but also during hyphal growth. Although mechanical force has been reported as an inducer of mitochondrial fission in animal cells, such fission is still dependent on Drp1 (Helle et al., 2017). However, it has also been reported that human cell infection by *Listeria monocytogenes* can trigger Drp1-independent fission of the mitochondrial network (Carvalho et al., 2020). Conidiation in *A. nidulans* might represent an interesting model to dissect DnmA -dependent and -independent mechanisms of mitochondrial fission. Because the lack of DnmA or FisA does not drastically affect peroxisome number in glucose media, we consider that *ΔdnmA* and *ΔfisA* developmental defects are caused mainly by the lack of mitochondrial division.

We have also shown that the induction of mitochondrial division by H_2_O_2_ is a DnmA and FisA-dependent process and not an unspecific fragmentation process, as reported in *Podospora anserina*, where Dnm1-independent mitochondrial fragmentation was observed in the presence of H_2_O_2_ and in senescent cultures *dnm1* or *fis1* deletion did not affect mycelial morphology, growth rate or fertility but resulted in a notable increase in life span (Scheckhuber et al., 2007). However, DNM1 and FIS1 have recently been shown to be required for *P. anserina* normal ascospore development (Navarro-Espíndola et al., 2020).

In other fungi, mitochondrial morphology has been related to ROS levels and pathogenicity (Verma et al., 2018). In a notable example, the most virulent strains of the human pathogen *Cryptococcus deuterogattii* contain tubular mitochondria, and this correlates with an enhanced intracellular macrophage parasitism. Moreover, in these species H_2_O_2_ does not induce mitochondrial fission but rather mitochondrial tubularization (Voelz et al., 2014), indicating that despite these opposite outcomes, H_2_O_2_ plays a critical role in the regulation of mitochondrial dynamics.

In the plant pathogen *Ustilago maydis*, *dnm1Δ* mutants produce reduced disease symptoms in the plant when compared to those infected by the wild type (Mahlert et al., 2009). In *Magnaporthe oryzae* mitochondrial fragmentation occurs during infection and MoDnm1 is necessary for pathogenicity. The fact that during infection this fungus is exposed to high ROS levels, suggests that H_2_O_2_ produced by the plant could induce mitochondrial fission. Carbon starvation has been proposed as the trigger of mitochondrial fragmentation under these conditions (Kou et al., 2019). However, both factors might contribute to this process.

Although the lack of DnmA or FisA produced similar phenotypes, these proteins appear to have different functions under specific conditions (i.e., the utilization of different carbon sources). Different DnmA and FisA functions have been reported in *U. maydis*, where the overexpression of *lga2* induces mitochondrial fragmentation and mitophagy in a process that depends on Dnm1 but not on Fis1, despite the fact that *dnm1Δ* and *fis1Δ* mutants display similar mitochondrial fission defects during growth (Nieto-Jacobo et al., 2012). Interestingly, the *a2* mating-type locus *lga2* gene encodes a mitochondrial protein critical for uniparental mitochondrial DNA inheritance during sexual development, which links this process to mitochondrial fission. This suggests that the sexual defects we observe in *ΔdnmA* and *ΔfisA* mutants could be related to defects in uniparental mitochondrial DNA inheritance.

There is strong evidence showing that ROS regulate fungal development (Hansberg and Aguirre, 1990; Lara-Ortiz et al., 2003; Aguirre et al., 2005; Cano-Dominguez et al., 2008, 2019; Lara-Rojas et al., 2011; Mendoza-Martinez et al., 2017; Garrido-Bazan et al., 2018). We have shown that mitochondrial division is critically associated to fungal development and ROS production and that mitochondrial division is in turn regulated by ROS. Additional research is needed to determine the mechanisms that regulate these interrelated processes.

### Mitochondrial Function in Fission Defective Mutants

We detected that the lack of mitochondrial fission results in a moderate decrease of respiration *in vivo*. This is consistent with the decrease in cytochrome *c* oxidase activity detected in isolated mitochondria from *A. fumigatus ΔdnmA*, and *ΔfisA* mutants (Neubauer et al., 2015). Respiration might be affected by the type of external carbon source available. Notably, the *ΔdnmA* mutant presented a more normal appearance and conidiation when grown in glycerol as sole carbon source. Glycerol utilization in fungi involves a FAD-dependent glycerol 3-phosphate dehydrogenase located at the outer surface of the inner mitochondrial membrane, which via FADH2 directly transfers electrons to the respiratory chain (Klein et al., 2017). How exactly is mitochondrial fission related to respiration remains to be determined.

### Mitochondrial Morphology Is Related to Mitochondrial ROS Production

Our results indicate that mitochondrial shape affects ROS production. Indeed, mitochondria from *ΔdnmA* and *ΔfisA* mutants were readily stained by MitoSOX in conditions where WT mitochondria were not stained. Two results support that it is superoxide what we are detecting in our MitoSOX experiments. First, pretreatment of *ΔdnmA* and *ΔfisA* mutants with the mitochondrial superoxide scavenger mito TEMPO drastically decreased MitoSOX staining. Second, *ΔdnmA* and *ΔfisA* mutants are sensitive to the mitochondrial superoxide inducing compound menadione, but not to H_2_O_2_. Interestingly, WT mitochondria are also stained by MitoSOX when the WT strain is grown on ethanol as sole carbon source. We have shown that ethanol induces oxidative stress, as determined by the nuclear localization NapA, a redox-regulated transcription factor (Mendoza-Martinez et al., 2017). More recently, it has been shown that ethanol causes skeletal muscle mitochondrial dysfunction, which is reversed by mito TEMPO (Kumar et al., 2019), suggesting that ethanol also induces mitochondrial superoxide formation in animal cells.

Notably, *ΔdnmA* and *ΔfisA* mitochondria are not homogenously stained by MitoSOX but rather some regions clearly show higher fluorescence spots that change in position during observation, suggesting dynamic changes in the concentrations of ROS along non-dividing mitochondria. This is reminiscent of the stochastic and intermittent bursts of superoxide that have been described in respiratory mitochondria from animal cells (Hou et al., 2014), as such mitoflashes are also exacerbated by decreased mitochondrial fission (Li et al., 2016; Zhang et al., 2017). Despite their high mitochondrial ROS levels, *ΔdnmA*, and *ΔfisA* mutants appear adapted to oxidative stress, as indicated by their ability to relocalize the SrkA kinase to the nucleus in response to high H_2_O_2_.

While mitochondrial fission has been generally associated with cell division, damage and mitophagy, our results suggest that cellular ROS, produced by different cellular activities, including respiration, might regulate mitochondrial division and this in turn regulate mitochondrial ROS production.

## Data Availability Statement

All datasets generated for this study are included in the article/Supplementary Material.

## Author Contributions

All authors listed have made a substantial, direct and intellectual contribution to the work, and approved it for publication.

## Conflict of Interest

The authors declare that the research was conducted in the absence of any commercial or financial relationships that could be construed as a potential conflict of interest.
