# Supplementary material for: DnmA and FisA Mediate Mitochondria and Peroxisome Fission, and Regulate Mitochondrial Function, ROS Production and Development in Aspergillus nidulans
Source: Front Microbiol. 2020 May 4;11:837. doi: 10.3389/fmicb.2020.00837 (PMC7232558; doi:10.3389/fmicb.2020.00837)
Supplement: Supplementary file 1 [file Data_Sheet_1.PDF]

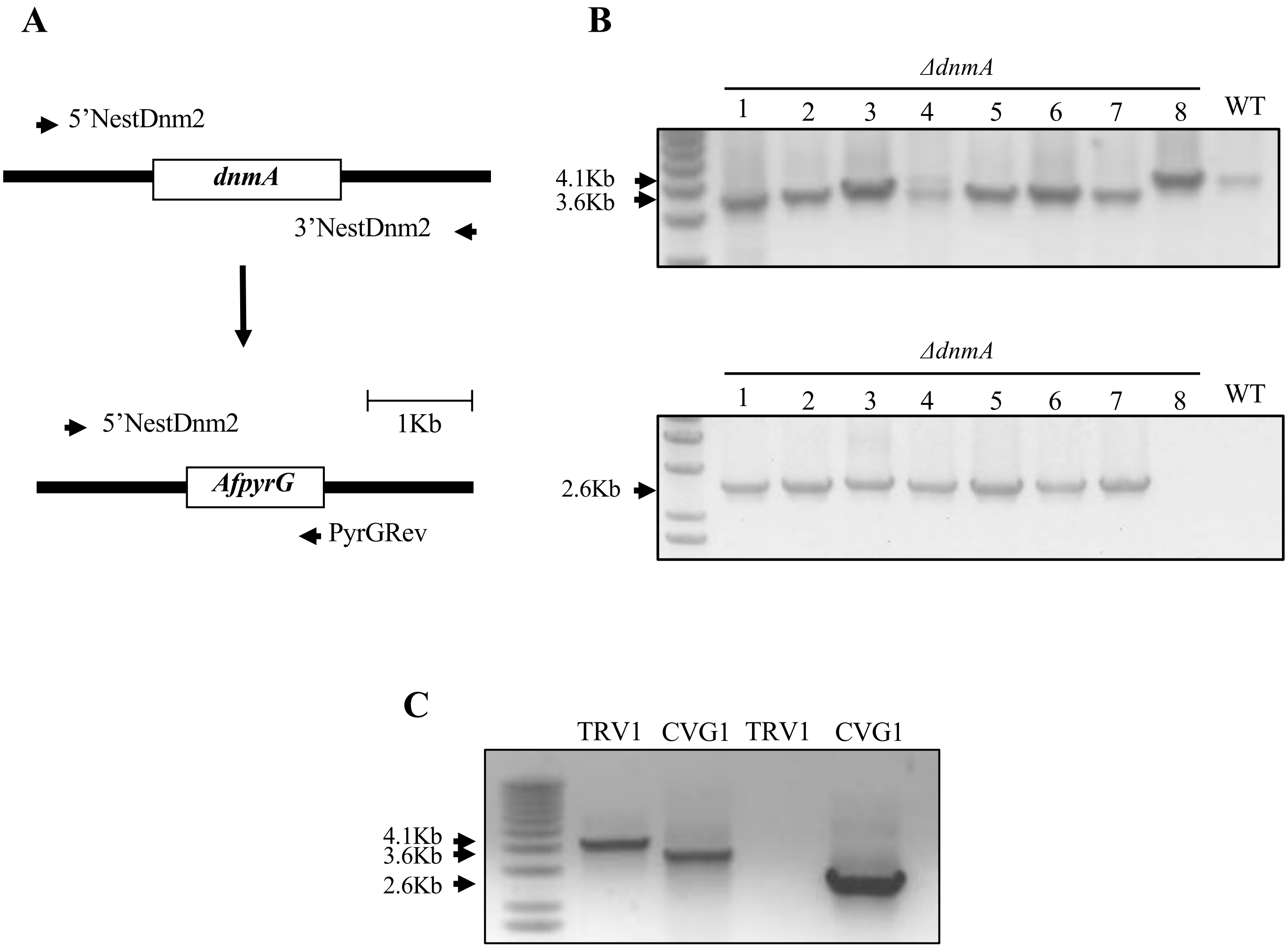

FIGURE S1. Deletion of the *dnmA* gene. (**A**) A *dnmA* deletion construct, containing *AfpyrG* gene as selective marker, was used to transform strain A1155. DNA from selected transformants 1-8 was used to confirm *dnmA* deletion by PCR, using primers 5'NestDnm2 and 3'NestDnm2. These generate 3.6 and 4.1 Kb products in *ΔdnmA* and WT strains, respectively. **(B)** *dnmA* deletion in transformants 1-8 was further confirmed by a second PCR using primers 5'NestDnm2 and PyrGRev, which generate a 2.6 Kb product. Transformant 2 was renamed TVG1 and used in further experiments. TVG1 was crossed with strain TRV1, containing mitochondria labeled with mCherry, and progeny with labeled mitochondria was analyzed for *dnmA* deletion. (**C**) DNA from strains TRV1 and CVG1 was used in PCR reactions with primers 5'NestDnm2 and 3'NestDnm2, which generate 3.6 (*ΔdnmA)* and 4.1 Kb (TRV1) products (left lanes). Right lanes show a 2.6 Kb PCR product generated only for strain CVG1 (*ΔdnmA)* with primers 5'NestDnm2 and PyrGRev. *ΔdnmA* strain CVG1, containing labeled mitochondria, was chosen and used in further experiments.

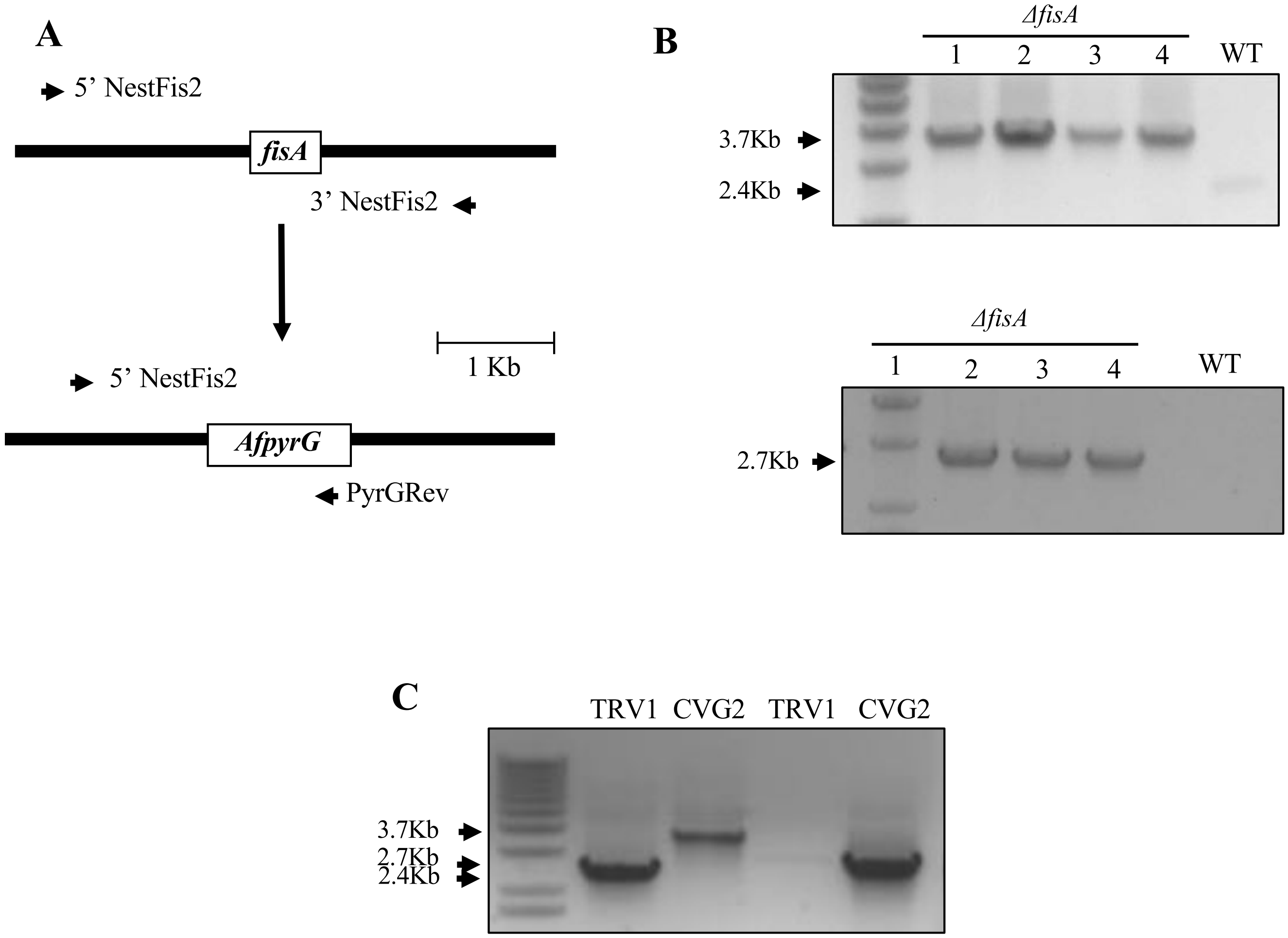

FIGURE S2. Deletion of the *fisA* gene. (A) A *fisA* deletion construct containing *AfpyrG* gene as selective marker, was used to transform strain A1155. DNA from transformants 1-4 was used as template for PCR with primers 5’NestFis2 and 3’NestFis2, which generate 2.4 and 3.7 Kb products in WT and *ΔfisA* strains. (**B)** A second PCR with primers 5’NestFis2 and PyrGRev, which generate a 2.7 Kb product, was used to confirm *fisA* deletion. Transformant 3 was selected, renamed as TVG2 and used in further experiments. TVG2 was crossed with strain TRV1, containing mitochondria labeled with mCherry, and a *ΔfisA* progeny strain with labeled mitochondria was named as CVG2 and chosen for further studies. (**C**) DNA from strains TRV1 and CVG2 was used in PCR reactions with primers 5’NestFis2 and 3’NestFis2, which generate 3.7 (*ΔfisA)* and 2.4 Kb (TRV1) products (left two lanes). Right lanes show a 2.7 Kb PCR product generated only for strain CVG2 (*ΔfisA),* with primers 5’NestFis2 and PyrGRev.

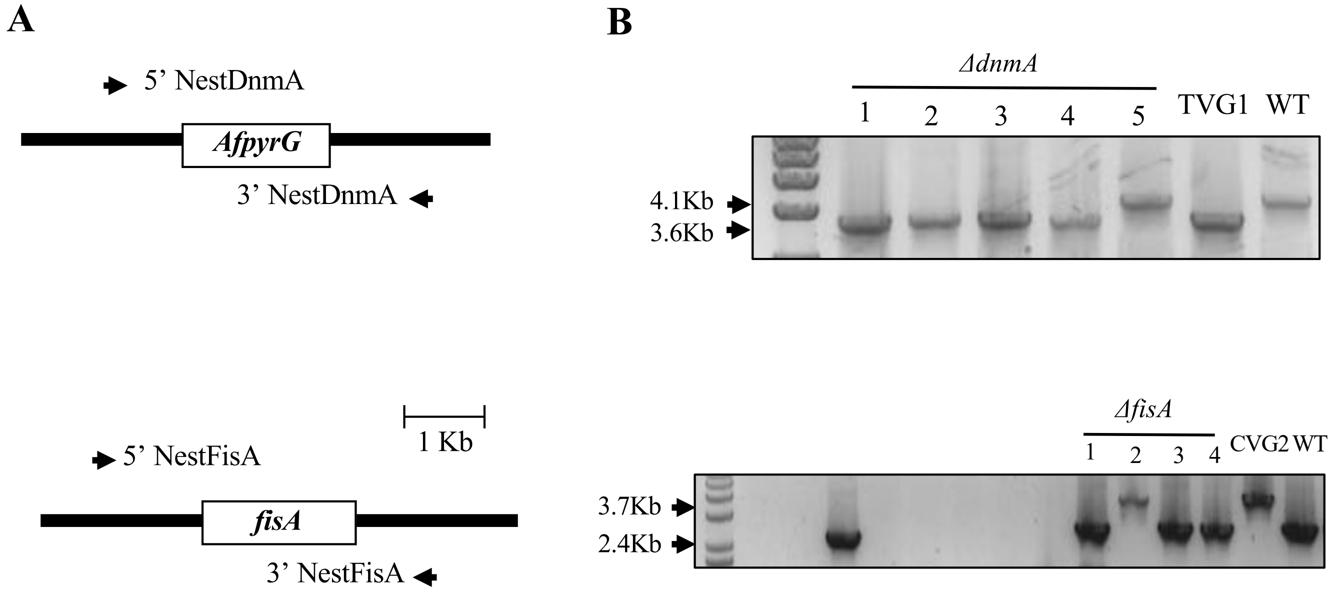

FIGURE S3. PCR confirmation of *ΔdnmA ΔfisA* double mutants. (A) DNA from selected progeny from a TVG1 X CVG2 cross was used as template for PCR, using primers 5'NestDnmA and 3' NestDnmA, which generates 3.6 and 4.1 Kb products in *ΔdnmA* and WT strains, respectively. **(B)** *fisA* deletion was confirmed using primers 5' NestFisA and 3'NestFisA, which generate a 3.7 and 2.4 Kb products in *ΔfisA* and WT strains, respectively. Strain 2 was chosen as *ΔdnmA ΔfisA* double mutant and used in other experiments.

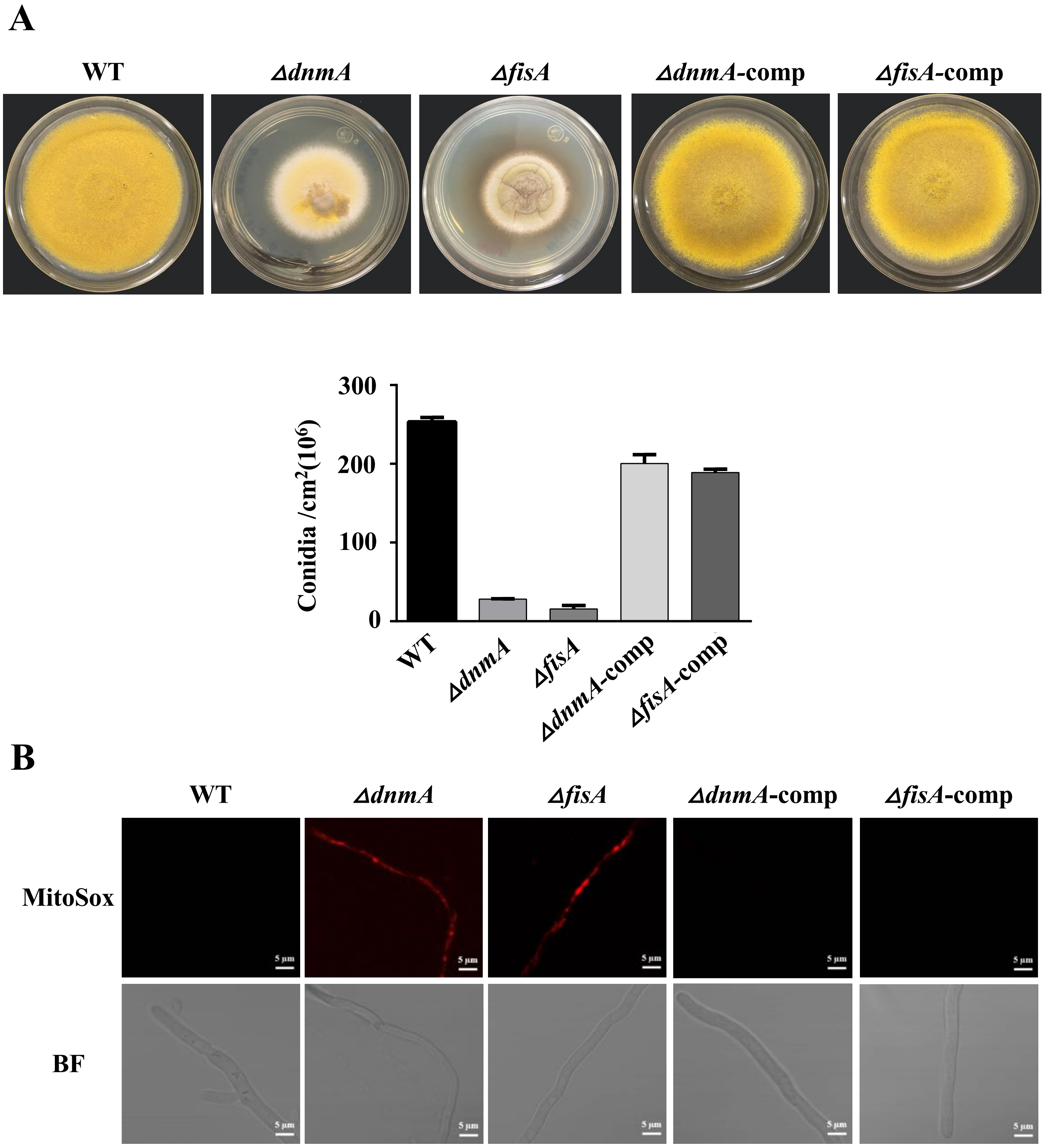

FIGURE S4. Genetic complementation of Δ*dnmA* and Δ*fisA* mutants. (**A**) Strains CVG36 (Δ*dnmA* Δ*argB*) and CVG37 (Δ*fisA* Δ*argB*) were transformed with *argB* plasmids pVDnmA and pVFisA, respectively. Conidia ($1x10^4$) from trains RMS011 (WT), CVG36, CVG37, CVG38 (Δ*dnmA*-comp) and CVG39 (Δ*fisA*-comp) were inoculated on supplemented glucose-MM plates, incubated at 37ºC during 6 days and conidia number determined (lower panel). Bars indicate standard deviation from three independent experiments. (**B**) The same strains were stained with MitoSox and observed using confocal microscopy. BF = Bright field.

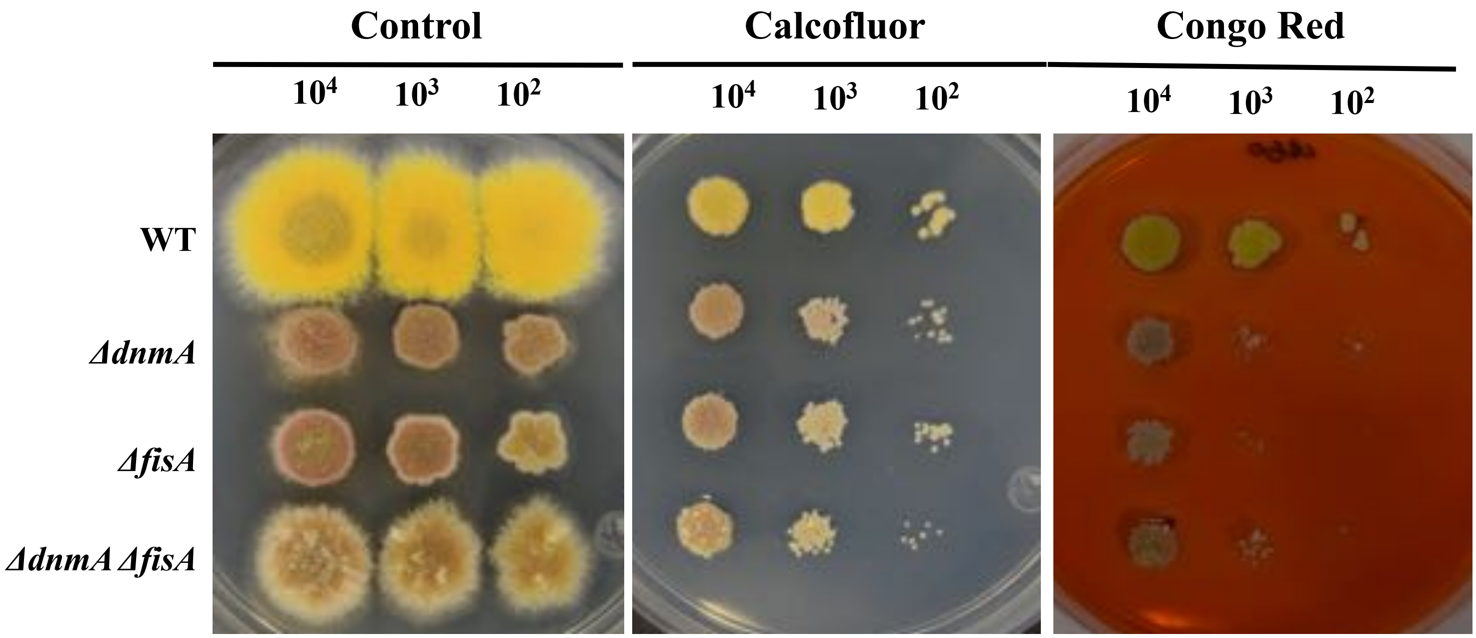

FIGURE S5. Mutants Δ*dnmA* and Δ*fisA* are not sensitive to calcofluor but do show sensitivity to congo red. Spores from strains TRV1 (WT), CVG1 (*ΔdnmA*), CVG2 (*ΔfisA*) and CVG3 (Δ*dnmA* Δ*fisA*) were inoculated on supplemented MM plates containing cell wall-damaging compounds calcofluor (30 µg/ml) and congo red (50 µg/ml), and incubated at 37ºC during 2 days.

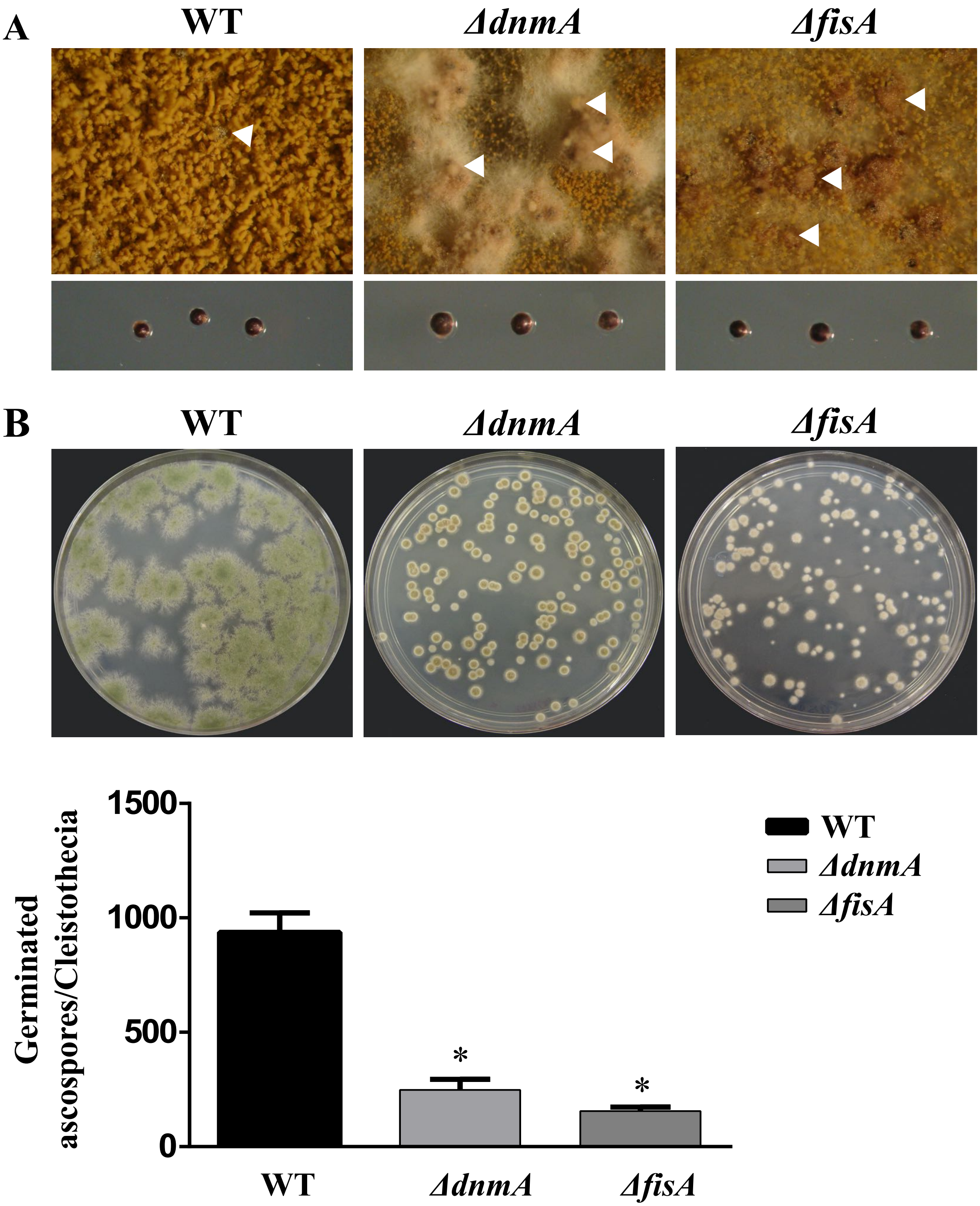

Figure S6. ***ΔdnmA* and *ΔfisA* mutants show increased formation of Hülle cells and develop cleitothecia with a decreased ascospore content. (A)** Strains WT (TRV1), CVG1 (*ΔdnmA*) and CVG2 (*ΔfisA*) were induced to undergo sexual development as reported (Kawasaki et al., 2002). Pictures of confluent cultures were taken after eight days of induction. White arrowheads indicate aggregates of Hülle cells. Lower panel shows cleitothecia isolated from the indicated strains. **(B)** Isolated cleistothecia from strains WT (A1155), TVG1 (*ΔdnmA*) and TVG2 (*ΔfisA*) were crushed in 500, 50 and 50 µl of H_2O_2, respectively, and 50 µl of each ascospore suspension were plated on supplemented minimal medium, incubated at 37 ºC for four days, and the number of colonies determined. Top panel shows a representative experiment. Lower panel shows total germinated ascospores per cleistothecia as the media from three independent experiments. Bars indicate standard deviation. Data analyzed by one-way ANOVA, following Tukey's test (*$p < 0.05$). Asterisks indicate significant differences with respect to the WT strain.

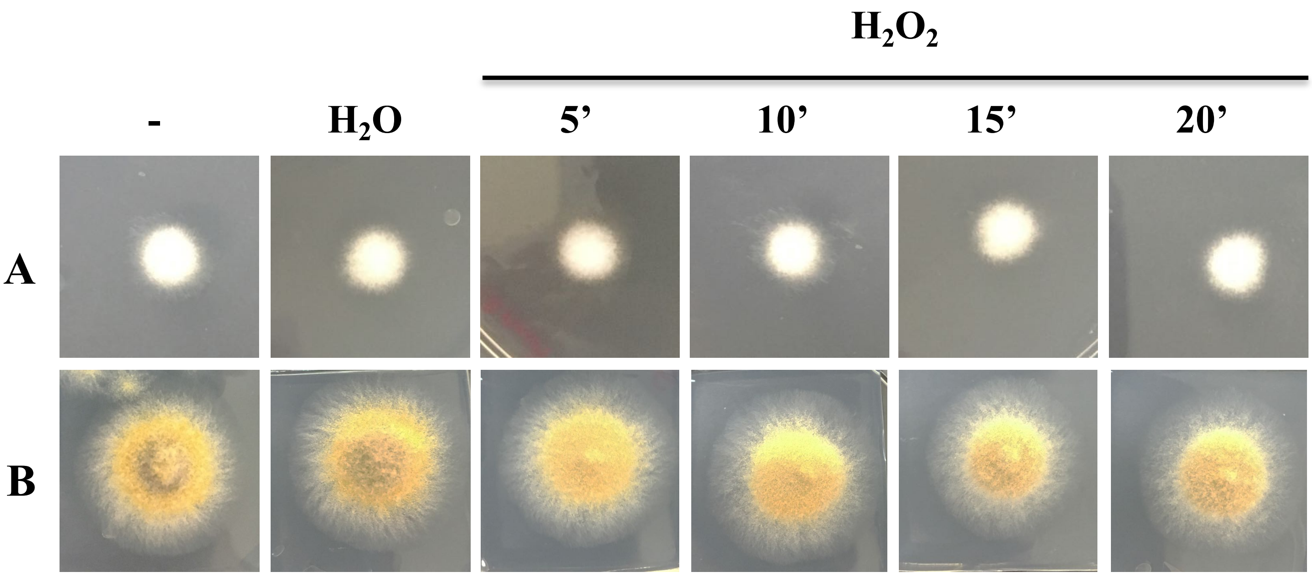

FIGURE S7. *A. nidulans* growth is not affected by a transient treatment with 5 mM hydrogen peroxide. (A) Conidia (1X10^4) from wild type strain TRV1 were used to inoculate supplemented MM and incubated at 37 ºC for 14 h and then colonies were covered with a 5 mM H_2O_2 solution for the indicated times and then removed. **(B)** After H_2O_2 treatment, colonies were re-incubated at 37 ºC for 24 hours.

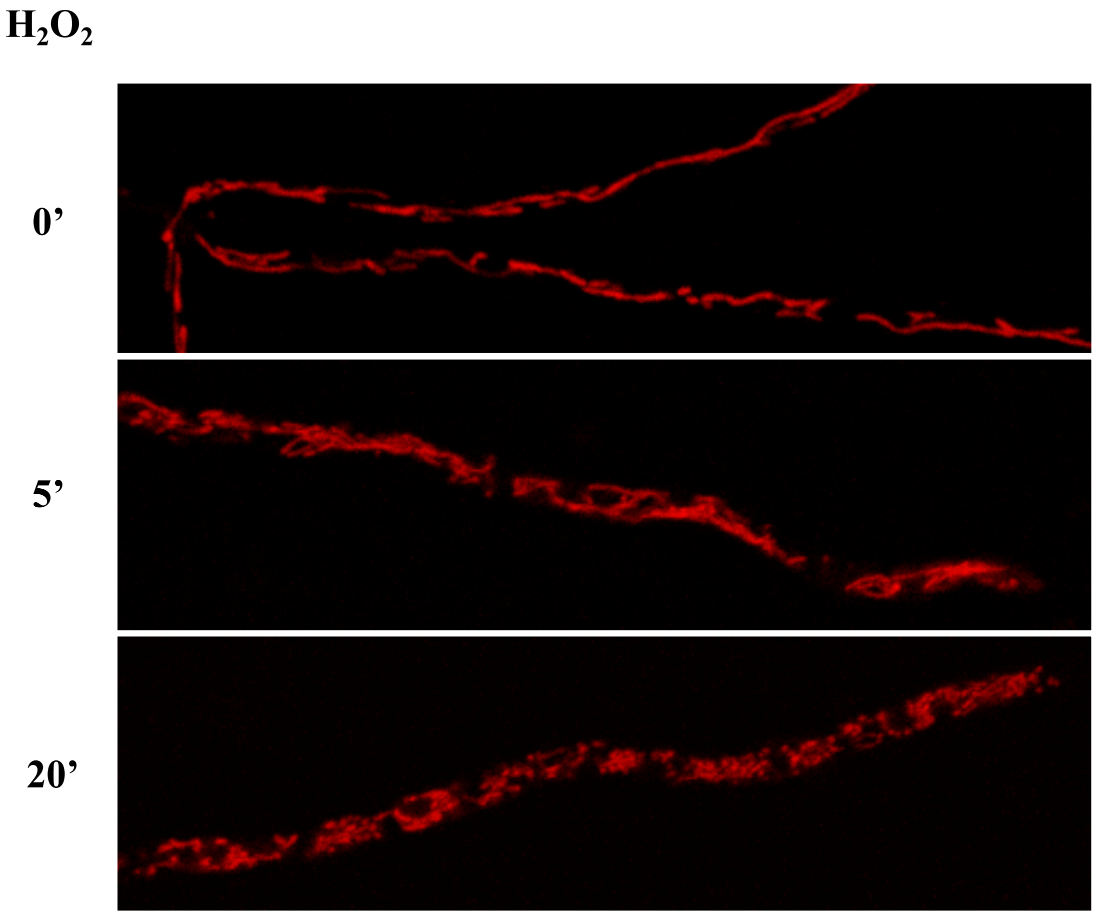

Figure S8. Hydrogen peroxide induces mitochondrial fission in *A. nidulans*. Strain TRV1, containing mitochondria labeled with mCherry, was grown for 14 h and treated with 5 mM H_2O_2 as indicated in **Figure S6** for 0, 5 and 20 min and observed using confocal microscopy.

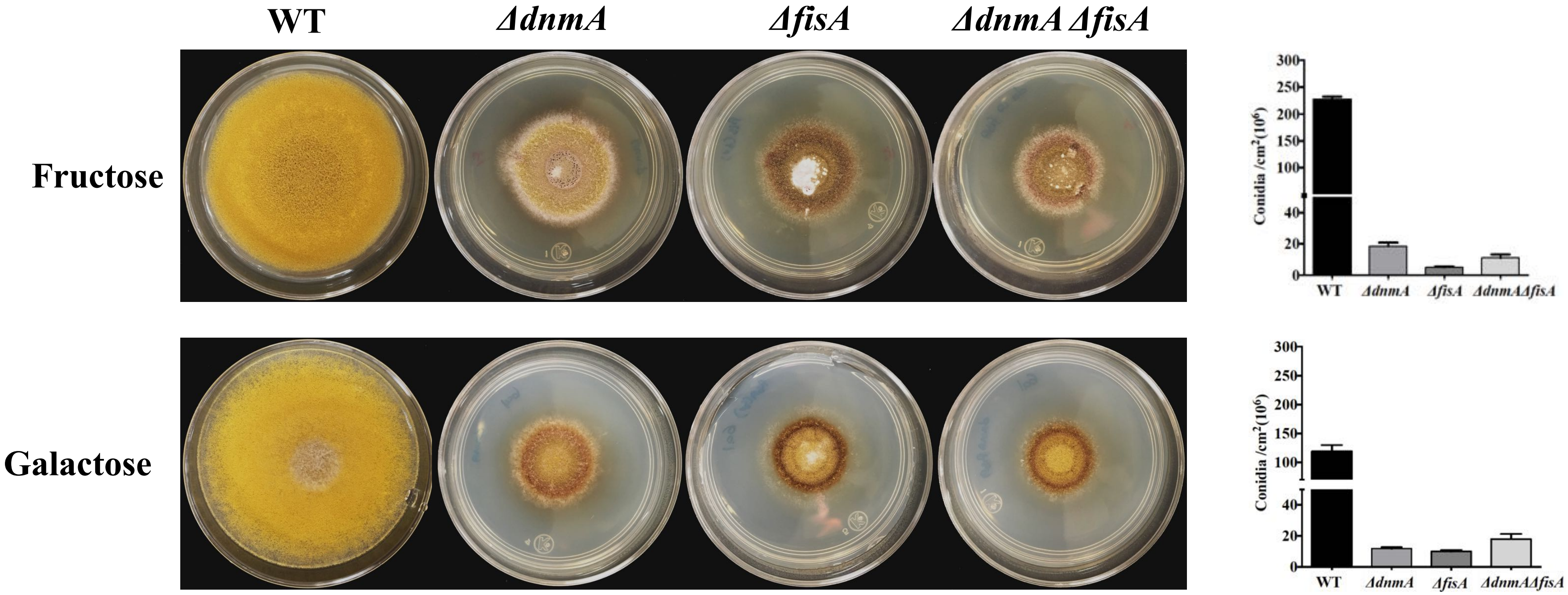

FIGURE S9. Growth and conidiation of Δ*dnmA,* Δ*fisA* and Δ*dnmA* Δ*fisA* mutants in fructose or galactose as sole carbon sources. Spores from strains TRV1 (WT), CVG1 (*ΔdnmA*), CVG2 (*ΔfisA*) and CVG3 (Δ*dnmA* Δ*fisA*) were inoculated on supplemented MM plates containing the indicated carbon sources and incubated at 37 ºC for 3 days. After this, total conidiospores per colony were harvested, counted and the count divided by colony area to determine number of conidia per square centimeter. Bars indicate standard deviation from three independent experiments.

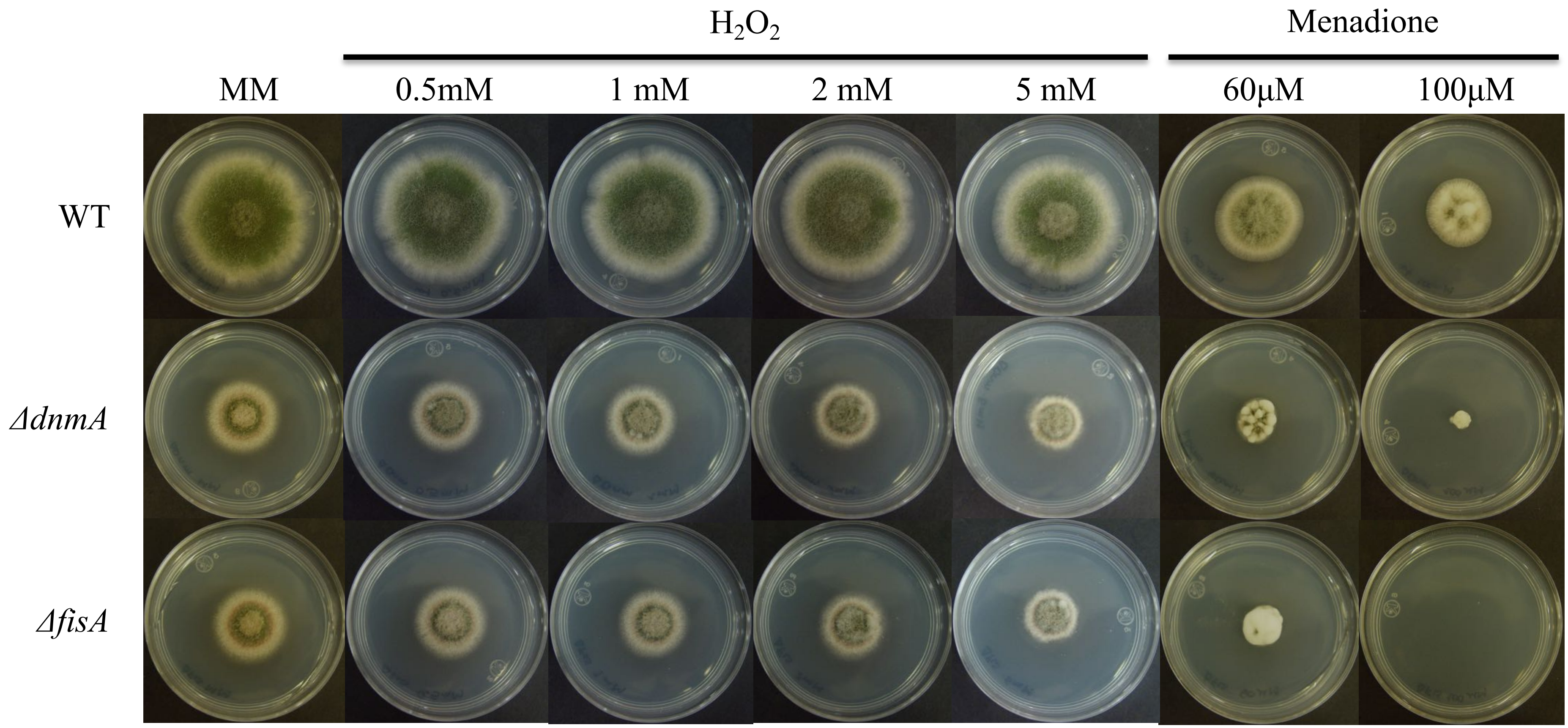

FIGURE S10. Δ*dnmA* and Δ*fisA mutants* are not sensitive to H_2O_2 but do show sensitivity to menadione. Spores from strains 1155 (WT), TVG1 (*ΔdnmA*) and TVG2 (*ΔfisA*) were inoculated on supplemented MM plates containing H_2O_2 or menadione, at the indicated concentrations, and incubated at 37ºC for 3 days.
