# Supplementary material for: DnmA and FisA Mediate Mitochondria and Peroxisome Fission, and Regulate Mitochondrial Function, ROS Production and Development in Aspergillus nidulans
Source: Front Microbiol. 2020 May 4;11:837. doi: 10.3389/fmicb.2020.00837 (PMC7232558; doi:10.3389/fmicb.2020.00837)
Supplement: Supplementary file 2 [file Data_Sheet_2.docx]

**Table S1**. *Aspergillus nidulans* strains used in this study

| Strain | Genotype | Source |
| --- | --- | --- |
| CLK43 | *pabaA1 yA2 veA1* | Kawasaki *et al.,* (2002) |
| 11035 | *pyrG89 pyroA4 riboB2* ∆*nkuA::argB veA1* | M. Hynes; Nayak *et al*., (2006) |
| 1155 | *pyrG89 pyroA4* ∆*nkuA*::*bar veA1* | Fungal Genetics Stock Center |
| TRV1 | *pabaA1 yA2 veA1 gadph (p)::atp9-7(mts)::mcherry veA1* | This work: CLK43 transformed with pPABLE |
| TVG1 | *pyrG89 pyroA4 ∆dnmA::AfpyrG ∆nkuA::bar veA1* | This work;  1155 transformed with PCR construct dnmA-AfpyrG-dnmA |
| TVG2 | *pyrG89 pyroA4 ∆fisA::AfpyrG ∆nkuA::bar veA1* | This work;  1155 transformed with PCR construct fisA-AfpyrG-fisA |
| CVG1 | *pabaA1 yA2 ∆dnmA::AfpyrG gadph (p)::atp9-7(mts)::mcherry veA1*^a^ | This work; progeny  from TRV1 X TVG1 |
| CVG2 | *pabaA1 yA2 ∆fisA::AfpyrG gadph (p)::atp9-7(mts)::mcherry veA1*^a^ | This work; progeny  from TRV1 X TVG2 |
| CVG4 | *pabaA1 yA2 ∆dnmA::AfpyrG srkA::GFP::AfpyrG gadph (p)::atp9-7(mts)::mcherry veA1*^a^ | This work; progeny  from TRJ1 x CVG1 |
| CVG5 | *pabaA1 yA2 ∆dnmA::AfpyrG ∆fisA::AfpyrG gadph (p)::atp9-7(mts)::mcherry veA1*^a^ | This work; progeny  from TVG1 X CVG2 |
| RPA | *yA::[gpdA(p)-mcherry-FLAG-PTS1::Afpyro], [TagGFP2::rabA::AfpyrG], riboB2, pyroA4, pyrG89, pabaA, nkuA:: argB+ veA1* | Tan et *al.,*(2014) |
| CVG30 | *pabaA1 yA2 ∆dnmA::AfpyrG yA::[gpdA(p)-mcherry-FLAG-PTS1::Afpyro] veA1* | This work; progeny  from RPA X TVG1 |
| CVG31 | *pabaA1 yA2 ∆fisA::AfpyrG yA::[gpdA(p)-mcherry-FLAG-PTS1::Afpyro] veA1* | This work; progeny  from RPA X TVG2 |
| TRJ1 | *pyrG89 pyroA4 srkA::GFP ∆nkuA::bar veA1* | Jaimes-Arroyo et *al*., (2015) |
| RMS011 | *pabaA1 yA2 ∆argB::trpC∆B trpC801 veA1* | M. Stringer |
| CVG36 | *pabaA1 yA2 ∆argB::trpC∆B ∆dnmA::pyrG trpC801 veA1^a^* | This work; progeny from RMSO11 X TVG1 |
| CVG37 | *pabaA1 yA2 ∆argB::trpC∆B ∆fisA::pyrG trpC801 veA1*^a^ | This work; progeny from RMSO11 X TVG2 |
| CVG38 | *pabaA1 yA2 ∆dnmA::pyrG argB::dnmA veA1^a,b^* | This work; CVG36 transformed with pVDnmA plasmid |
| CVG39 | *pabaA1 yA2 ∆fisA::pyrG argB::fisA veA1^a,b^* | This work; CVG37 transformed with pVFisA plasmid |

^a^It may contain *pyrG89*

*^b^*It may contain *∆argB::trpC∆B trpC801*

**Table S2**. DNA primers used in this study

| Primer | Sequence (5′ to 3′) |
| --- | --- |
| 5'ForDnm2 | CAGGCATCTCAGGCAGACTTCAA |
| 5’NestDnm2 | TGACGAGCTCTGCGGAACGAT |
| 5'RevDnm2 | GAGGGTGAAGAGCATTGTTTGAGGCACAGGTTTTCCCCCAAGGCG |
| 3'ForDnm2 | GCATCAGTGCCTCCTCTCAGACACATAACGCCTGTCTGTCCGTG |
| 3'RevDnm2 | CATCCGCGCGAGACTCTTTCG |
| 3'NestDnm2 | GCGGAGTGCATCTTCTCTCCG |
| PyrGFor | GCCTCAAACAATGCTCTTCACC |
| PyrGRev | GTCTGAGAGGAGGCACTGATGC |
| 5'ForFis 2 | GCGTTGAAGTCGAAGATTGGGC |
| 5'NestFis 2 | CATCTCCCTTGCGGCTGTTACG |
| 5'RevFis 2 | GAGGGTGAAGAGCATTGTTTGAGGCATTGCTGGAATACAGTACCACTGTCT |
| 3'ForFis2 | GCATCAGTGCCTCCTCTCAGACAAATGCAGGGTAATAGGGTGCTGAG |
| 3'RevFis2 | GGTTGTGGTCTGCAAAGGGAT TC |
| 3'NestFis2 | GGAACCAGTGTGTGCAAGGAAGC |
| CV1 | GGTACCCAGCTTTTGTTCCC |
| CV2 | TCCACTAGTTCTAGAGCGGCC |
| CV3 | TCTAGAACTAGTGGAAGGCGGTCGGCTGGGGTAG |
| CV4 | CAAAAGCTGGGTACCTGCATCTTCTCTCCGCGAAATCG |
| CV5 | TCTAGAACTAGTGGAATAGATAAACAAAAGCAAAGATCCA |
| CV6 | CAAAAGCTGGGTACCTCATCTTGCTGTACACGATAAGACT |
